# Group B *Streptococcus* transcriptome when interacting with brain endothelial cells

**DOI:** 10.1128/jb.00087-24

**Published:** 2024-05-21

**Authors:** Nadine Vollmuth, Bailey E. Bridgers, Madelyn L. Armstrong, Jacob F. Wood, Abigail R. Gildea, Eric R. Espinal, Thomas A. Hooven, Giulia Barbieri, Alexander J. Westermann, Till Sauerwein, Konrad U. Foerstner, Alexandra Schubert-Unkmeir, Brandon J. Kim

**Affiliations:** 1 Department of Biological Sciences, University of Alabama, Tuscaloosa, Alabama, USA; 2 Department of Pediatrics, University of Pittsburgh School of Medicine, Pittsburgh, Pennsylvania, USA; 3 Richard King Mellon Institute for Pediatric Research, UPMC Children’s Hospital of Pittsburgh, Pittsburgh, Pennsylvania, USA; 4 Department of Biology and Biotechnology, University of Pavia, Pavia, Italy; 5 Institute of Molecular Infection Biology (IMIB), University of Wuerzburg, Wuerzburg, Germany; 6 Helmholtz Institute for RNA-based Infection Research (HIRI), Helmholtz Centre for Infection Research (HZI), Wuerzburg, Germany; 7 ZB MED, Information Centre for Life Sciences, Cologne, Germany; 8 TH Koeln, University of Applied Sciences, Cologne, Germany; 9 Institute for Hygiene and Microbiology, University of Wuerzburg, Wuerzburg, Germany; 10 Department of Microbiology, University of Alabama at Birmingham Heesink School of Medicine, Birmingham, Alabama, USA; 11 University of Alabama Center of Convergent Biosciences and Medicine, Tuscaloosa, Alabama, USA; 12 University of Alabama Life Research, Tuscaloosa, Alabama, USA; University of Illinois Chicago, Chicago, Illinois, USA

**Keywords:** Group B* Streptococcus*, *Streptococcus agalactiae*, blood–brain barrier, brain endothelial cells, transcriptome

## Abstract

Bacterial meningitis is a life-threatening infection of the central nervous system (CNS) that occurs when bacteria are able to cross the blood–brain barrier (BBB) or the meningeal–cerebrospinal fluid barrier (mBCSFB). The BBB and mBCSFB comprise highly specialized brain endothelial cells (BECs) that typically restrict pathogen entry. Group B *Streptococcus* (GBS or *Streptococcus agalactiae*) is the leading cause of neonatal meningitis. Until recently, identification of GBS virulence factors has relied on genetic screening approaches. Instead, we here conducted RNA-seq analysis on GBS when interacting with induced pluripotent stem cell-derived BECs (iBECs) to pinpoint virulence-associated genes. Of the 2,068 annotated protein-coding genes of GBS, 430 transcripts displayed significant changes in expression after interacting with BECs. Notably, we found that the majority of differentially expressed GBS transcripts were downregulated (360 genes) during infection of iBECs. Interestingly, *codY*, encoding a pleiotropic transcriptional repressor in low-G + C Gram-positive bacteria, was identified as being highly downregulated. We conducted qPCR to confirm the *codY* downregulation observed via RNA-seq during the GBS–iBEC interaction and obtained *codY* mutants in three different GBS background parental strains. As anticipated from the RNA-seq results, the ∆*codY* strains were more adherent and invasive in two *in vitro* BEC models. Together, this demonstrates the utility of RNA-seq during the BEC interaction to identify GBS virulence modulators.

**IMPORTANCE:**

Group B *Streptococcus* (GBS) meningitis remains the leading cause of neonatal meningitis. Research work has identified surface factors and two-component systems that contribute to GBS disruption of the blood–brain barrier (BBB). These discoveries often relied on genetic screening approaches. Here, we provide transcriptomic data describing how GBS changes its transcriptome when interacting with brain endothelial cells. Additionally, we have phenotypically validated these data by obtaining mutants of a select regulator that is highly down-regulated during infection and testing on our BBB model. This work provides the research field with a validated data set that can provide an insight into potential pathways that GBS requires to interact with the BBB and open the door to new discoveries.

## INTRODUCTION

Bacterial meningitis is an infection of the central nervous system (CNS) caused by pathogenic bacteria, which, without treatment, is inevitably lethal (1). Worldwide, bacterial meningitis is included in the top 10 causes of death due to infection (2). Group B *Streptococcus* (*Streptococcus agalactiae* or GBS) is an opportunistic pathogen capable of causing bacterial meningitis, among other conditions such as sepsis, pneumonia, and encephalopathy, in susceptible populations (3). This risk is particularly potent in neonates and is associated with asymptomatic colonization of the vaginal tract in as many as 15%–40% of pregnant mothers by GBS (4). Transmission to newborns portends significant increases in infant mortality and morbidity; in total, GBS poses the greatest risk of severe invasive infection in neonates and, in combination with *Escherichia coli,* leads to at least two-thirds of all neonatal meningitis-mediated deaths (4).

To cause meningitis, bacteria such as GBS must penetrate the blood–brain barrier (BBB) or the meningeal–cerebrospinal fluid barrier (mBCSFB). The BBB and mBCSFB are primarily composed of specialized brain endothelial cells (BECs) that serve as gatekeepers preventing entrance of pathogens to the brain via a variety of mechanisms, including tight junctions between cells (composed of characteristic proteins such as Occludin, ZO-1, and Claudin-5) and tightly regulated endocytosis and pinocytosis (5–7). Disruption of brain barriers is a hallmark of bacterial meningitis, and mechanisms of how BECs fail to protect the CNS during bacterial meningitis remain poorly understood (1, 2, 5, 7, 8).

A key challenge in determining molecular mechanisms of bacterial penetration of the CNS lies in the development and use of a human-based model that possesses BBB characteristics necessary for the study of disease progression. While *in vivo* models have the advantages of natural immune response and development of bacteremia, they can have limitations in the study of human-specific pathogens and may have important interspecies variation in BBB components that limit application to human health and disease (5, 9). Many *in vitro* immortalized BBB models lack phenotypes that would be present in a human BEC, including proper establishment of tight junctions (5, 9). These cell lines thus show markedly decreased transendothelial electrical resistance (TEER); additionally, they are limited in their interaction with other cells vital to barrier function. Brain-like endothelial cells generated from induced pluripotent stem cells (iBECs), in contrast, display the many expected phenotypes of a human BBB, including proper establishment of tight junctions between cells, physiological TEER values, and increased response to other cell types within the BBB microenvironment (10–13). Importantly, this model has shown great promise in the study of bacterial interactions with BECs by a number of viral and bacterial pathogens including GBS, supporting its utility in the investigation of GBS–BBB interactions (5, 14–20).

Transposon mutant libraries have allowed researchers to identify GBS virulence factors that contribute to GBS–BEC interactions (21). Using these genetic tools, factors such as invasion-associated gene (*iag*) (21) and the pilus locus with pili proteins such as *pilA*, *pilB*, and *pilC* (22), have been identified and characterized. However, identification of novel virulence factors can be limited to the readout of the screening method (such as invasion of BECs). To determine how GBS alters gene expression when interacting with BECs and potentially identify GBS regulatory genes or novel virulence factors, we performed RNA sequencing (RNA-seq) and differential gene expression analysis on GBS compared with GBS interacting with iBECs. We found that GBS alters its transcriptome when interacting with BECs. A total of 430 transcripts were found to significantly change in expression after interacting with the BECs of a total of 2,068 annotated protein-coding genes of GBS. Surprisingly, only 70 regulated genes exhibited transcriptional upregulation, whereas 360 differentially regulated genes showed transcriptional downregulation. We selected a highly downregulated gene, *codY,* for phenotypic validation of observed transcriptional changes. CodY is a global transcriptional regulator of Gram-positive bacteria known to alter the metabolic response to varying nutrient availability; interestingly, it has also been observed to repress virulence genes in pathogens such as *Streptococcus pyogenes* (23–27). Recently, the role of CodY in GBS pathogenesis was explored in two contexts. In GBS strains NCTC10/84 and A909, *codY* deletion mutants were associated with stringent response, and stringent response promoted GBS persistence and enhanced virulence in human blood (26). A central effector of the bacterial stringent response is RelA, which phosphorylates GTP to generate alarmone molecules guanosine tetra- and pentaphosphate [(p)ppGpp], leading to global transcriptional changes, and CodY activity is regulated by the balance of GTP and (p)ppGpp (26). Generation of a knockout strain of highly pathogenic BM110 revealed a decrease in *in vivo* pathogenicity and a reduced ability to adhere to epithelial cell monolayers *in vitro*, indicating its potential necessity to pathogenesis (27). In contrast, here, we find that ∆*codY* strains exhibit increased adherence and invasion in two different BEC *in vitro* models, providing a functional insight into the downregulation observed in the transcriptomic data. GBS adherence to epithelia versus endothelia has differences that may require variation in expression of cohorts of virulence factors. Together, these findings increase our knowledge of how GBS responds to BECs and provide an important resource to motivate further analysis of GBS virulence and physiology during infection.

## RESULTS

### RNA sequencing indicates transcriptome changes in GBS during infection

**Fig 1:**
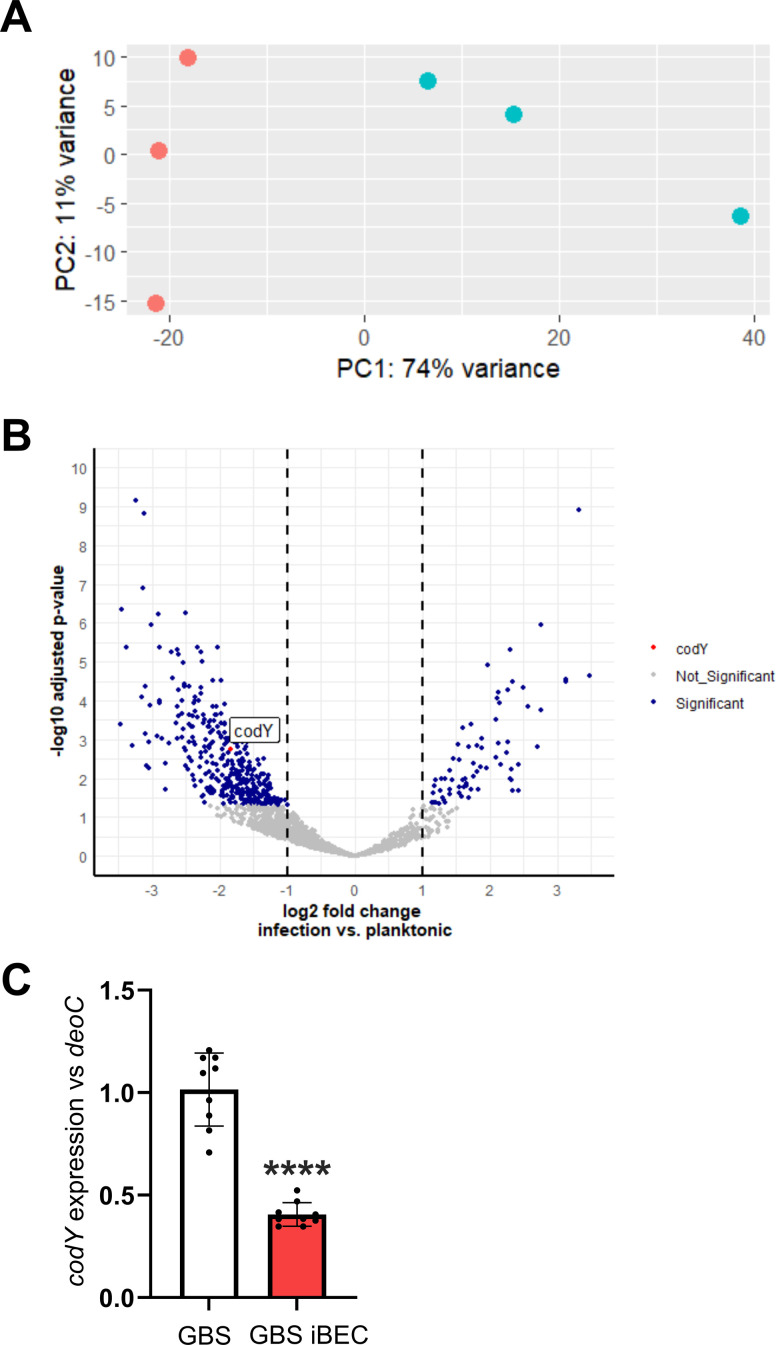
Altered GBS transcriptome during interaction with BECs. (**A**) Principal component analysis of the GBS transcriptome with or without interaction with BECs. Three individual biological replicates and color are represented with (red) or without BEC interaction (green). (**B**) Volcano plot describing up- and downregulated genes in the GBS transcriptome. Blue indicates an adjusted *P*-value < 0.05 (red is *codY*, and gray is not significant). The X-axis indicates log2 fold change infection versus planktonic, and the y-axis indicates -log10 adjusted *P*-value. (**C**) qPCR performed for *codY* and normalized to *deoC* for COH1 GBS strain and COH1 interacting with iBEC MOI of 10 for 5 hours. Data presented are from three biological replicates performed in triplicates. Students *t-*test (**C**), ****P < 0.0001.

To determine the changes that occur in GBS gene expression during infection of the human BBB, we performed triplicate RNA-seq of wild-type (WT) COH1 GBS during infection of induced pluripotent stem cell-derived BECs and in cell culture conditions in the absence of iBECs. In total, 24-wells with or without iBECs were infected with GBS at an MOI of 10 for 5 hours. Fresh EC medim (see Materials and Methods for details) was added to the wells right before the infection. Both groups were treated identical. The iBEC model was generated using established protocols, and immunofluorescence for common BEC markers was performed for confirmation (28, 29) (Fig. S1). Total RNA of the adherent iBECs and GBS or GBS only was isolated immediately after 5 hours (see Materials and Methods for details). Reads were normalized and mapped to an updated COH1 genome annotation that captures noncoding and coding sequences (30). Differential gene expression analysis revealed statistically significant changes in the bacterial transcriptome associated with the interaction of GBS with BECs (Fig. 1; Table S1). In total, 2,068 annotated protein-coding genes of GBS were detected, 430 of which displayed statistically significant changes in expression following interaction with the BECs (Fig. 1). Interestingly, 360 of differentially regulated genes exhibited transcriptional downregulation during the GBS–BEC interaction (Fig. 1). This finding suggests a marked bacterial transcriptional response to *in vitro* infection of iBECs.

### Relevance of codY downregulation is supported through increased adherence and invasion of *∆codY* mutant strains

**Fig 2:**
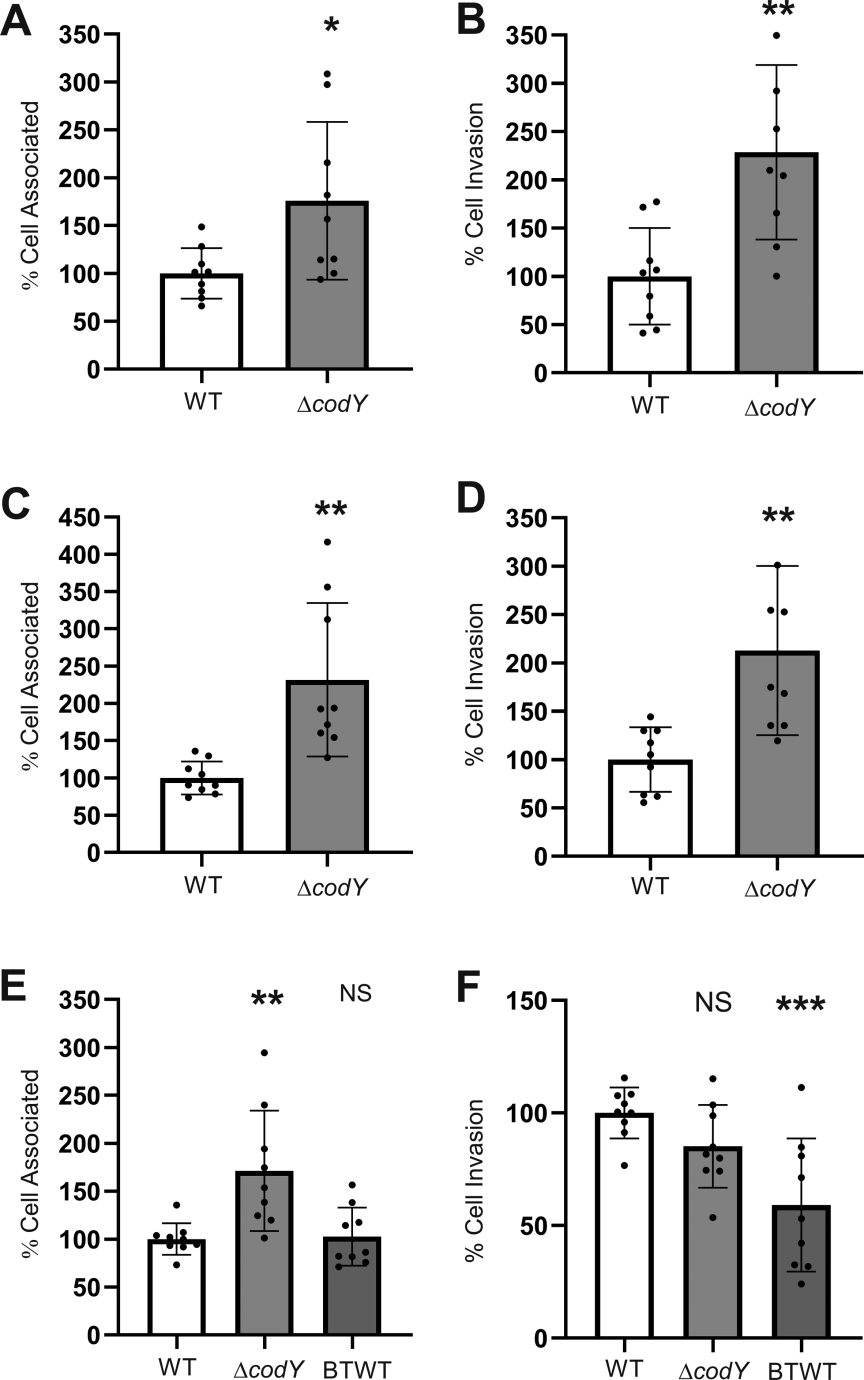
GBS attachment and invasion is enhanced on iBECs with Δ*codY* mutants. (**A-B**) GBS attachment and invasion is increased with Δ*codY* mutants in the A909 background strain compared to the WT. (**C-D**) Similarly, increased attachment and invasion is observed with the Δ*codY* NCTC 10/84 GBS background strain when compared to the WT. (**E**) The BM110 background strain Δ*codY* mutant shows increased attachment as well compared to the WT, and the complemented mutant (BTWT) reduced attachment back to the WT. (**F**) The BM110 background strain Δ*codY* mutant shows no change in invasion compared to the WT. All experiments were conducted in technical and biological triplicate, *n* = 9. (**A-D**) Student’s t-test was performed. (**E-F**) one-way ANOVA was performed, *P < 0.05, **P < 0.01, and ***P < 0.001.

**Fig 3:**
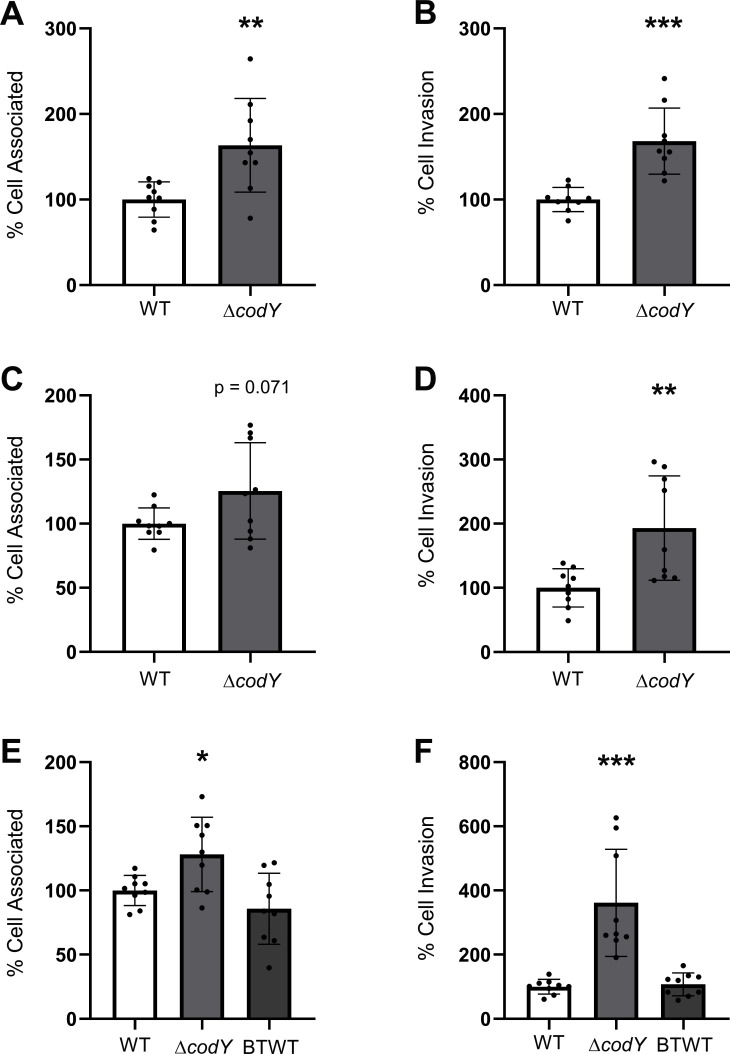
GBS attachment and invasion is enhanced on hCMEC/D3 cells with Δ*codY* mutants. (**A-B**) GBS background A909 attachment and invasion with Δ*codY* is significantly increased compared to that of the WT strain. (**C-D**) NCTC 10/84 Δ*codY* GBS exhibited a trend for increased attachment and a significant increase in invasion when compared to the WT. (**E-F**) BM110 background strain Δ*codY* mutant attachment and invasion is significantly increased, and the complemented mutant (BTWT) shows attachment and invasion comparable to those of the WT. Experiments were performed in technical and biological triplicate, *n* = 9. (**A-D**) Student’s *t* test was performed and (**E-F**) one-way ANOVA. *P < 0.05, **P < 0.01, and ***P < 0.001.

One of the downregulated genes with high significance (*P* = 0.001828) codes for the pleiotropic transcriptional repressor CodY. We conducted qPCR to confirm downregulation of *codY* during the interaction of GBS with iBECs (Fig. 1C). To functionally assess these findings, we obtained ∆*codY* mutants and parent WT in three different GBS background strains (26, 27) and tested the adherence and invasion in the iBEC (Fig. 2) and hCMEC/D3 (Fig. 3) *in vitro* models. We anticipated that as *codY* is highly downregulated during adherence of GBS WT to iBECs, a ∆*codY* mutant would be associated with elevated adherence and invasion rates *in vitro*. Indeed, all three ∆*codY* strains exhibited enhanced adherence, and all except BM110 in iBECs but only in hCMEC/D3 displayed increases in invasion compared to the isogenic WT strains (Fig. 2 (A–B: A909, C–D: NCTC 10/84, E–F: BM110) and Fig. 3 (A–B: A909, C–D: NCTC 10/84, E–F: BM110)). For BM110 belonging to serotype III, MLST-17 strains with the same sequence type as COH1, we additionally obtained the chromosomally complemented strain (BTWT), which restored the adherence and invasion levels similar to those of the WT strain (Fig. 2E, 3E and F). Together, these findings suggest an increase in bacterial attachment and ability to invade human BBB models following decreased *codY* expression. These data provide proof-of-principle phenotypic validation to the RNA-seq differential gene expression analysis and suggest CodY as a virulence suppressor in BEC-interacting GBS.

## DISCUSSION

Knowledge of the mechanism of penetration of the human BBB by pathogenic Group B *Streptococcus* during bacterial meningitis is crucial to understand the ways that the BBB fails to protect the CNS during serious infection. GBS invasive disease remains widespread, with GBS causing 86% of cases of bacterial meningitis in infants < 2 months of age, of which 11% of cases were fatal (31). GBS disease burden is much higher in low- and middle-income countries in which intrapartum antibiotic prophylaxis following screening for GBS colonization of the maternal genital tract is less accessible; among countries with such protocols, the burden of early-onset GBS disease and other morbidities associated with GBS are greatly diminished (3, 32). Nevertheless, the global disease burden is such that further investigation is necessary to understand the infection process more fully and, in doing so, more effectively treat and prevent GBS-mediated neonatal meningitis.

One vital component of the infection process that remains incompletely elucidated is the manner in which bacterial gene expression changes in response to interaction with the human BBB. Here, a transcriptomic approach was employed to determine how GBS altered its gene expression when interacting with BECs. We performed RNA-seq-based comparative analysis of the bacterial transcriptome during infection of an iBEC BBB model. This allowed for the identification of a wide variety of transcripts, both well-characterized and as yet undescribed, that are differentially expressed during GBS infection. In total, 2,068 annotated protein-coding genes of GBS were detected both in culture and during infection of iBECs (Fig. 1; Table S1). The resultant bacterial genetic response was striking, showing widespread transcriptome alteration in response to interaction with iBECs.

In this study, we provided proof-of-concept support of our differential gene expression analysis. We hypothesized that complete genetic knockout of a highly downregulated gene in GBS may further enhance the pathogen’s ability to interact with BECs. The gene selected was *codY*, encoding a repressor previously known to contribute to virulence in many Gram-positive bacteria and enhance GBS survival in blood. Its role in GBS was only recently elucidated (23–27). Although downregulated in our data set, *codY* has been reported as a required gene for GBS virulence, with ∆*codY* strains demonstrating decreased pathogenesis in human epithelial cervix adenocarcinoma (HeLa) and human epithelial lung carcinoma (A549) cell lines as well as mouse models (27). Additional motivation for selecting *codY* is supported by our transcriptomic data, as *codY* is known to repress genes of the *arc* operon (33, 34). In our data set, we also observed that lower expression of *codY* was correlated with upregulation of the *arc* operon (Table S1). Of note, our data show an upregulation trend for phospho/transferases and metabolism supporting enzymes for transport and metabolism of sugars and other carbon sources besides the *arc* operon. It has been shown that transporters for carbohydrates such as glucose, galactose, and mannose impacted invasive pneumococcal disease (35). Interestingly, previously described adhesins (like Srr2 and pili) remained unchanged in our transcriptomic data. This suggests that either these described adhesins are already being expressed, or our data set, selecting for adherent bacteria, only sequenced bacteria that were expressing these adhesins. Regardless, this data set may uncover novel adhesins or GBS factors that contribute to the interaction with BECs. Moreover, we confirmed the reduced *codY* expression during the interaction of GBS with iBECs via qPCR (Fig. 1C). After obtaining ∆*codY* mutants in three background strains of GBS, we tested the ability of these mutants compared to the WT to interact with BECs *in vitro* and found the ∆*codY* mutants to have enhanced adherence and invasion (Fig. 2 and 3). Previous work has showed that the strain NCTC10/84 has a single-nucleotide insertion in an intergenic homopolymeric tract being responsible for an unusually strong hemolytic activity (36). Despite that, *codY* was still behaving as expected in our experiments. These findings suggest that adherence of GBS to epithelia compared to endothelia has differences that may require variation in the expression of cohorts of virulence factors.

These findings support our RNA-seq findings and their use in future investigation of GBS pathogenesis during human CNS infection. This transcriptomic data set provides a valuable resource for the GBS scientific community in the search for novel virulence factors that may contribute to GBS infection in the human BBB. The identification of novel mechanisms of GBS circumvention and/or destruction of the brain barriers protecting the CNS may offer a better understanding of disease progression, allowing for the possible development of novel therapies or methods to prevent GBS meningitis-associated morbidity and mortality.

## MATERIALS AND METHODS

### Maintenance and differentiation of iBECs

Differentiation of induced pluripotent stem cells (iPSCs) into brain-like endothelial cells was conducted as previously described (12, 14, 28). iPSC cell line IMR90/C4 (WiCell) was grown in StemFlex medium (Thermo Scientific) changed daily and maintained on Matrigel (Fisher Scientific) -coated plates (VWR). For differentiation, iPSCs were lifted off the well with Accutase (StemCell Technologies) into a single-cell suspension. The single-cell suspension was seeded at a density of 10,000 cells/cm^2^ onto Matrigel-coated flasks and grown for 3 days in StemFlex medium. To initiate differentiation, the StemFlex medium was removed by aspiration and replaced with UM medium [Dulbecco’s modified Eagle medium (DMEM), F12 medium 1:1 (Gibco)] plus 20% knockout serum replacement [Gibco], 1% minimal essential medium [Gibco], 0.5% Glutamax [Gibco], and 0.07% beta-mercaptoethanol [VWR] for 6 days. Media were replaced daily. After 6 days in UM medium, the differentiation is changed into the EC medium [human endothelial cell serum-free medium (Gibco) plus 1% B27 supplement (Gibco)] plus 20 ng/mL basic fibroblast growth factor (Peprotech) and 10 µM all-trans retinoic acid (Sigma) for 2 days. iBECs were then purified onto a fibronectin–collagen IV-coated plate for infection assays and immunofluorescence or a transwell insert for monitoring of transendothelial electrical resistance (TEER). Cells were changed into EC media without all-trans retinoic acid or basic fibroblast growth factor the following day. TEER readings were measured, and analysis for expression of BEC markers was performed for each differentiation as an internal quality control.

### Immunofluorescence

Cells were fixed with ice-cold methanol, blocked with 10% FBS in PBS, and stained for PECAM (CD-31), Glut1, P-glycoprotein (P-gp) (Thermo Fisher), Occludin, Claudin-5, ZO-1 (Invitrogen), and VE-cadherin (Santa Cruz), as previously described (28, 29). Images were taken with the Nikon Ti2 microscope.

### Bacterial strains and growth conditions

Group B *Streptococcus* (GBS; *Streptococcus agalactiae*) strain COH1 [serotype III, multi-locus sequence type 17 (MLST-17)] (37), NCTC10/84 (serotype V, MLST-26) (38), A909 (serotype Ia) (39), and BM110 (serotype III, MLST-17) (40, 41) were used for these studies (list of strains Table 1). *ΔcodY* mutants were previously characterized and utilized for validation experiments (26, 27). RNA-seq was conducted using the COH1 wild-type strain. Wild-type GBS strains and BM110 *ΔcodY* mutant were cultivated in Todd–Hewitt broth (THB) at 37°C in static culture. NTCT10/84 and A909 *ΔcodY* mutants were cultivated in THB +5 µg/mL erythromycin at 37°C in static culture.

**TABLE 1:** Strains used in this study

Strain	Source	Antibiotic
COH1 [serotype III, multilocus sequence type 17 (MLST-17)]	(37)	None
NCTC10/84 (serotype 252 V, MLST-26)	(38)	None
A909 (serotype Ia)	(39)	None
BM110 (serotype III, MLST-17)	(40, 41)	None
NCTC10/84 ΔcodY	(26)	5 µg/mL erythromycin
A909 ΔcodY	(26)	5 µg/mL erythromycin
BM110 ΔcodY	(27)	None
BTWT (BM110 ΔcodY chromosomally complemented strain)	(27)	None

### Growth of hCMEC/D3 cell line

hCMEC/D3 cells were maintained on tissue culture flasks coated with 1% rat tail collagen in EndoGRO MV medium (Millipore-Sigma). Cells were split onto 24-well tissue culture plastic dishes coated with 1% rat tail collagen for experiments and allowed to grow to confluency (4 days) before infection experiments were conducted.

### GBS infection and RNA sequencing

iBECs were differentiated as described above onto a coated 24-well tissue culture plate and infected with GBS at an MOI of 10 for 5 hours, which has previously been demonstrated to not have cytotoxic effects (17). Before infection, the media were changed to fresh human endothelial cell serum-free (EC) media without all-trans retinoic acid or basic fibroblast growth factor. For the GBS control group, the wells without iBECs got at the same time fresh EC media without all-trans retinoic acid or basic fibroblast growth factor, and GBS was added the same way as to the iBEC well. After 5 hours of incubation, the medium was removed from the wells and discarded. Cells were then washed 1 x in PBS, and total RNA was immediately collected from cells and any adherent bacteria using the mirVana RNA (Thermo Scientific) isolation kit, and libraries were prepared and verified on a Bioanalyzer (Agilent RNA 6000 Nano Kit, assay mode Prokaryote Total RNA Nano, Agilent Technologies). Three independent differentiations and three independent biological experiments were conducted for the generation of libraries for sequencing (~2×10^5 iBEC cells per experiment). For Illumina sequencing, libraries were pooled in equimolar concentrations and fractionated using a differential clean-up with the Agencourt AMPure kit. Sequencing was performed on a NextSeq500 platform (Illumina) in the single-end mode for 75 cycles.

### Computational analysis of transcriptomic data

First, Illumina reads were trimmed using cutadapt (versions 1.15) (42). Illumina’s TruSeq “Read 1” adapter sequence was removed from the 3’ end. Nucleotides with a Phred quality score lower than 20 and their following downstream (5’ to 3’) bases were cut. Further filtering steps and read mapping and downstream analysis, i.e., gene quantification and differential gene expression analysis, were done by the RNA-seq tool READemption (version: 0.4.3, DOI: 10.5281/zenodo.250598) (43). Further read filtering included clipping of poly(A) sequences and discarding of reads that had a read length below 20 nucleotides after performing the trimming steps. The read mapping was done using the short read mapper segemehl (version: 0.2.0) (44), which is integrated into READemption. The mapping was performed with a mapping accuracy of 95% and segemehl’s realigner lack (45). The human genome and annotation were obtained from GENCODE (version: 26, NCBI assembly name: GRCh38.p10) and the *Streptococcus agalactiae* COH1 genome and annotation from NCBI’s GenBank database (accession number: HG939456.1, Genbank assembly accession number: GCA_000689235.1). The *Streptococcus agalactiae* annotation was extended with sRNA entries taken from a previous publication (30). The gene quantification files, i.e., the number of reads overlapping with an annotated feature, were created using READemption. Afterward, the file was split up by species. Differential gene expression analysis and principal component analysis (PCA) were performed with the R package DESeq2 (version: 1.18.1, R version: 3.4.4) based on raw read countings (PCA vst−transformed counts) (46). Genes with an adjusted (Benjamini–Hochberg-corrected) *P*-value of 0.05 were defined as differentially expressed. The bioinformatical workflow is available under the DOI https://doi.org/10.5281/zenodo.10723304.

### RNA isolation and qPCR

iBECs were differentiated and infected as described for RNA sequencing. Using the mirVana RNA isolation kit (Thermo Scientific), total RNA was collected immediately after 5 hours of infection. cDNA was synthesized with the qScript cDNA Synthesis kit (Quantabio). SYBR Green (PowerUp SYBR Green Master Mix, Thermo Fisher) qPCR was conducted for GBS *codY* (GBSCOH1_RS07970), forward primer 5′-CCACCACCATAAATTGGAGCG-3′, and reverse primer 5′-GGTGGTGGTAACTTACTTGGC-3′. *deoC* (GBSCOH1_RS09680) was used as the housekeeping gene, forward primer 5′-CAGCAACATGGCCAGAAATC-3′, and reverse primer 5′-TTGACGTAAGAGGCTGGAATAC-3′. qPCR data were collected on the QuantStudio3 system (Applied Biosystems), and data are presented as fold change using the delta–delta–CT calculation (47).

### GBS infection assays

Monolayers of hCMEC/D3 or iBECs were cultured on 24-well plates. Adherence and invasion assays were conducted as previously described (17, 48–51). Overnight cultures of GBS were prepared the day before in THB at 37°C. The following day, GBS was subcultured into fresh THB and grown to an OD_600_ of 0.4. GBS was spun, washed, and resuspended in PBS OD_600_ of 0.4 and diluted in EC media without all-trans retinoic acid or basic fibroblast growth factor to achieve an MOI of 10 for GBS strains COH1, A909, and BM110 or MOI 0.1 strain NCTC 10/84. For adherence assays, cells and GBS were incubated at 37°C + 5% CO_2_ for 30 minutes. Cells were then washed 5 x in PBS to remove any non-adherent bacteria, removed from the plate with trypsin (10 minutes 0.25% trypsin–EDTA (1 x), VWR), lysed (0.025% Triton X-100), and diluted in PBS and plated onto THA (Todd–Hewitt agar) plates. Plates were incubated overnight at 37°C, and colonies were enumerated the following day. For invasion assays, cells were infected with GBS as above and incubated for 2 h followed by addition of 100 µg/mL gentamycin and incubated for an additional 2 h at 37°C + 5% CO_2_. Cells are then washed 3 x with PBS to remove antibiotics and plated onto THA plates for enumeration. For cell-associated and cell invasion %, we used the formula $\frac{\#CFU*dilution\;correction*volume\;correction}{Input\;CFU/well}$ and normalized to the percent of the WT. The input was determined for each individual experiment.

### Statistics

GraphPad Prism (version 9.1.2) was used for statistical analysis. *P* values of less than 0.05 were accepted as statistically significant. Student’s *t*-test was used for two-group comparisons; otherwise, one-way ANOVA was used.

## Data Availability

The data have been deposited in NCBI’s Gene Expression Omnibus (GEO) (52) and are accessible through GEO Series accession number GSE197489.

## SUPPLEMENTAL MATERIAL

Fig. S1 jb.00087-24-s0001.tif iBEC markers.

Supplemental legends jb.00087-24-s0002.docx Legends for Fig. S1 and Table S1.

Table S1 jb.00087-24-s0003.xlsx RNAseq data.

ASM does not own the copyrights to Supplemental Material that may be linked to, or accessed through, an article. The authors have granted ASM a non-exclusive, world-wide license to publish the Supplemental Material files. Please contact the corresponding author directly for reuse.
