# Supplementary figures and images for: Group B Streptococcus transcriptome when interacting with brain endothelial cells
Source: J Bacteriol. 2024 May 21;206(6):e00087-24. doi: 10.1128/jb.00087-24 (PMC11332166; doi:10.1128/jb.00087-24)

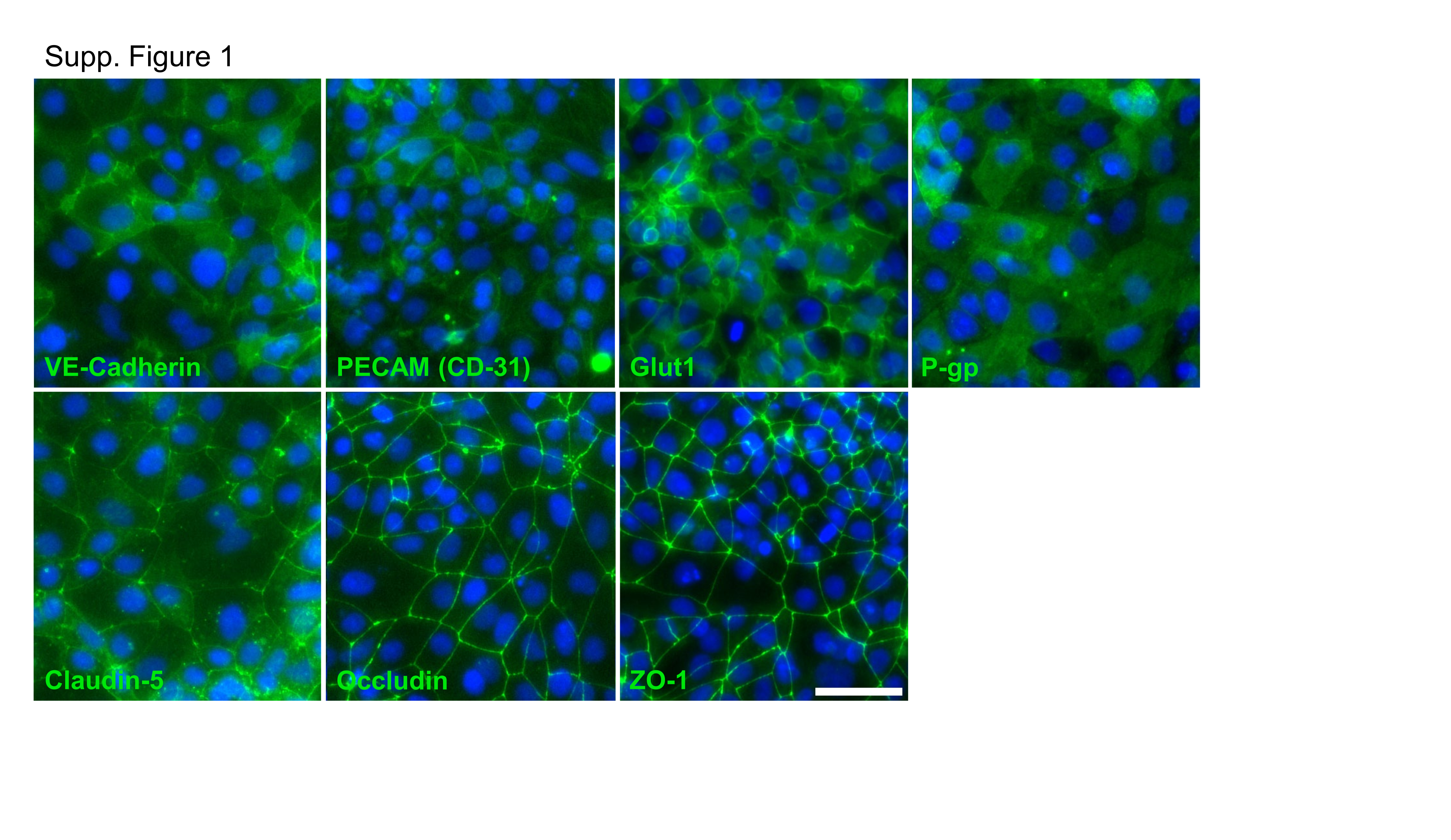

Supplement: Fig. S1 — iBEC markers. [file jb.00087-24-s0001.tif]
