# Supplementary material for: Group B Streptococcus transcriptome when interacting with brain endothelial cells
Source: J Bacteriol. 2024 May 21;206(6):e00087-24. doi: 10.1128/jb.00087-24 (PMC11332166; doi:10.1128/jb.00087-24)
Supplement: Supplemental legends — Legends for Fig. S1 and Table S1. [file jb.00087-24-s0002.docx]

**Supplement Figure Legends**

**Supplementary Figure 1: iBEC model characterization.** Immunostaining of endothelial markers PECAM (CD-1) and VE-Cadherin; transporters Glut1 and P-glycoprotein (P-gp); and tight junctions Occludin, Claudin-5 and Zona Occludin-1 (ZO-1). DAPI is shown in blue and all other markers in green. Scale bar = 50 µm.

**Supplementary Table 1: DEseq2 transcriptomic results.**
